# “RA.DI.CA.” Splint for the Management of the Mandibular Functional Limitation: A Retrospective Study on Patients with Anterior Disc Displacement without Reduction

**DOI:** 10.3390/ijerph17239057

**Published:** 2020-12-04

**Authors:** Carlo Di Paolo, Giovanni Falisi, Fabrizio Panti, Paola Di Giacomo, Alessandro Rampello

**Affiliations:** Gnathologic Division, Department of Oral and Maxillo-Facial Sciences, “Sapienza” University of Rome, Via Caserta 6, 00161 Rome, Italy; carlo.dipaolo@uniroma1.it (C.D.P.); g.falisi@tiscali.it (G.F.); fpanti@tiscali.it (F.P.); alessandro-rampello@alice.it (A.R.)

**Keywords:** disc displacement without reduction, jaw functional limitation, multifunctional splint, temporomandibular joint

## Abstract

The study aimed at assessing the effectiveness of the RA.DI.CA. splint in the management of temporomandibular joint disc displacement without reduction (ADDwoR) and jaw functional limitation. The authors developed a retrospective clinical study. A total of 2739 medical records were screened. One hundred and forty-one patients with chronic unilateral disc displacement without reduction and jaw limitation, treated with a multifunctional RA.DI.CA. splint, were enrolled. Temporomandibular pain, headache, familiar pain, neck pain, and emotional strain, maximum spontaneous mouth opening, and lateral excursions were evaluated at baseline (T0), after therapy (T1), and during the follow-up (T2). Descriptive statistical analysis was performed. Wilcoxon test assessed changes in symptomatology and functional aspects before and after treatment and between T1 and T2, with *p* < 0.05. Ninety-nine patients (70%) declared themselves “healed” from jaw functional limitation with no residual painful symptoms, 31 (22%) improved their symptoms and jaw function, 11 (8%) reported no changes compared to T0 and no one worsened. As for all parameters analyzed, the comparison between the ones before and after treatment was statistically significant (*p* < 0.05). The RA.DI.CA. splint proved to be highly performing and promoting functional and symptomatologic recovery, also in the medium and long term, through the restoration of the functional disc-condyle relationship and the healing of joint tissues.

## 1. Introduction

The most common issues that the gnathologist has to face in the management of temporomandibular disorders (TMD) are temporomandibular joint (TMJ) and/or masticatory muscles pain, as well as the qualitative and quantitative anomalies of the mandibular movements, due to the dysfunction of the stomatognathic components [1]. The alterations of the mandibular functionality are most frequently caused by the internal derangement of TMJ, with a prevalence of about 41% in TMD patients [2]. The main pathogenetic mechanism of these biomechanical joint disorders is the alteration of the functional relationship of the condyle-disc complex [3]. Joint disorders are characterized by a series of clinical, anatomical, and pathological clues, classified in the Diagnostic Criteria for Temporomandibular Disorders (DC/TMD), according to the degree and type of the TMJ dysfunction [4,5,6]. Among these, anterior disc displacement without reduction (ADDwoR) is an intra-capsular disorder with a prevalence of 5% [2], less frequent than disc displacement with reduction, characterized by restrictions in jaw movements, due to the permanent anterior displacement of the articular disc in relation to the condylar position [3]. The main signs and symptoms are quantitative and qualitative limitations of lateral, protrusive, and opening movements, accompanied by pain in the affected joint and less frequently in the contralateral one, during dynamic tasks and/or spontaneously [7]. Most patients seek for treatment when pain and jaw limitation interfere with daily activities, therefore reduction or elimination of pain and functional restriction are important parameters in evaluation of a therapeutic approach.

The course of this kind of pathology has been widely investigated, remaining a debated issue among clinicians. Some researchers believe that ADDwoR has a spontaneous resolution, at least under the aspect of the functional recovery [8]. Even if it is a self-limiting pathology, other researchers believe that it requires a proper diagnosis and early treatments in order to avoid future alterations in the soft and hard tissues of TMJ [9]. However, in accordance with the scientific literature [2], there are no further significant improvements after 12 weeks of natural course, so a conservative treatment is recommended.

Over the years, several therapeutic strategies have been proposed in scientific studies, classified in conservative and surgical treatments [4,5,10,11,12,13], accompanied by supportive therapies such as the pharmacological and physical ones [14]. Conservative therapies continue to be the most effective way to manage 90% of patients with TMD and surgery is the option of last resort if the previous ones fail [15].

The most debated conservative approach is the one using occlusal splints [9] and this is due to a huge variety of opinions about their specific action and design [5]. There are three types of occlusal splints which are commonly used in patients with jaw functional limitation: the stabilization, the distraction/pivot, and the anterior repositioning splints [16,17]. Many of them share common actions, however some are more suitable for the above-mentioned dysfunction.

In order to make therapeutic protocols as targeted as possible, the authors of the study have designed and patented a particular type of multifunctional splint, called RA.DI.CA. (acronym for Rampello–Di Paolo-Cascone License No. 91-000571, 3 September 1991), highly specific for jaw functional limitations. The characteristics of the device and its therapeutical effectiveness were evaluated in initial comparative studies [4,5,18,19,20].

The authors have been using it for years, adapting the protocols and some parts of the splint to the individual characteristics of the patient [18]. The choice of such device fits into a specific diagnostic path, which allows to select the therapy that is the most suitable and consistent with the patient’s condition [5,18].

In this retrospective clinical research, the authors aim at reporting the experience of the last decade in the use of RA.DI.CA splint in patients with ADDwoR and jaw limitation, and at monitoring the results of the application of this device, over the years, with a medium-long term follow-up.

## 2. Materials and Methods

### 2.1. Study Design

To address the research purpose, the authors developed and implemented a retrospective clinical research between January and December 2019. The study was approved by the Institutional Ethics Committee of Sapienza, University of Rome, Protocol no. 349.

Medical records of patients, taken in between 2007 and 2017 at the Gnathology Service, Department of Oral and Maxillo-facial Sciences, Sapienza University of Rome, were screened.

Patients who fulfilled the following criteria were enrolled in the study: (a) patients over 18 years old; (b) diagnosis of jaw functional limitation due to unilateral disc displacement without reduction for more than 6 months, verified in MRI (Magnetic Resonance Imaging), according to RDC/TMD Research Diagnostic Criteria for Temporomandibular Disorders) [3,18,19] and DC/TMD criteria [6]; (c) application of the RA.DI.CA. splint; (d) patients who had completed the therapy at least for 1 year; (e) at least 1-year follow-up.

The exclusion criteria were: (a) incomplete medical records; (b) jaw limitation due to other factors such as connective tissue diseases (sclerodermia), traumas (mandibular or condylar fractures), deformities, tumors, TMJ ankylosis, muscular locking; (c) other concomitant conservative therapies such as physical rehabilitation.

Out of 2739 medical records examined, 141 (5.14%) fulfilled the above inclusion and exclusion criteria.

All the patients were visited according to the RDC/TMD [3,21,22] until 2014 and later according to DC/TMD, evolution of the previous criteria [6]. The diagnosis and the resulting treatment plan were made on the basis of proper anamnesis, clinical evaluation, and TMJ imaging. All patients had signed the informed consent, according to the World Medical Association’s Declaration of Helsinki.

In order to collect a sample which was sufficiently large and homogenous by pathology, only patients with jaw limitation due to disc displacement without reduction were enrolled in the study, because ADDwoR is the most common cause of jaw limitation, compared to other pathologies.

Based on the analysis of the medical records, these categories of variables were considered.

1. Sociodemographic factors: gender, age, marital status, and occupation.
2. Occlusion: occlusal and skeletal class, dental formula, occlusal abnormalities, incisal guide, loss of teeth and parafunctions. These parameters were collected on the basis of the clinical examination and upon standard lateral radiograph and dental orthopantomography.
3. Medical History: previous trauma, previous click, and beginning of symptoms.
4. Types of pain: temporomandibular pain (arthralgia/muscle pain), headache, familiar pain, neck pain, and emotional strain, according to verbal numeric scale (VNS) before (T0) and after treatment—i.e., the end of the joint rehabilitation—(T1) and during the follow-up—i.e., patient monitoring after completion of therapy—(T2).
5. Functional aspects: maximum mouth opening and lateral excursions, expressed in mm before (T0) and after treatment (T1) and during the follow-up (T2).
6. Hours per day of splint application and duration of therapy.
7. Number of days necessary to resolution of closed-lock and degree of resolution.
8. Presence of click after the treatment and perception of any occlusal change.

In order to prevent systematic errors (bias), the authors used standardized protocols for data collection, including training of study personnel and blinding of operators during the evaluation of post-treatment patients’ status. Patients were selected using rigorous criteria to avoid confounding and evaluated according to validated international diagnostic criteria.

### 2.2. Sample Calculation

Sample size was calculated using the following statistical formula at 95% confidence interval:

$$n=\frac{1.96^{2}*0.05*\left(1-0.05\right)}{0.05^{2}},$$

where *n* = sample size; in the numerator, 0.05 is the expected prevalence and in the denominator, 0.05 is the level of required precision. The minimum sample size should have been of 73 subjects.

### 2.3. Data Collection

As for the mandibular functional aspects, maximum mouth opening was dichotomized as: no limitation (≥35 mm) and limitation (<35 mm). Lateral excursive movements and protrusive movements were categorized as: no limitation (≥6 mm) and limitation (<6 mm). As for pain symptomatology, data were collected on the basis of the values conferred upon it in terms of VNS (verbal numeric scale) and divided into the following categories: mild pain (0–29); moderate pain (30–60); severe pain (61–100).

As for the aspects of joint functionality and symptomatology at the end of therapy (T1) and during the follow-up (T2), results of treatment were expressed through the following categories:

T1 
  **H:**  **healed**, no sign, no symptom.
  **I:**  **improved**, absence of initial dysfunctional signs and symptoms with occasional presence of slight symptomatology and no symptoms worsened.
  **S:**  **stationary**, no change, neither improvement or worsening, of the previous state.
  **W:**  **worsened**, at least one symptom or one sign worsened and none improved.
T2 
  **H:**  **healed**,complete disappearance of the symptomatology present at T1.
  **I:**  **improved**, disappearance of at least one sign or symptom present at T1.
  **S:**  **stationary**, no change, neither improvement or worsening, of the previous state.
  **W:**  **worsened**, worsening of at least one symptom compared to T1.

### 2.4. Statistical Analysis

All analyses were performed with JASP Version 0.8.0.1, downloadable at https://jasp-stats.org/download/. Descriptive statistical analysis (percentage, average, median, mode, standard deviation, minimum, and maximum value) was performed for each variable of the study, collected at the baseline (T0), at the end of therapy (T1), and during the follow-up (T2).

Wilcoxon signed-rank test, which is a non-parametric statistical test for repeated measures on a single sample, was performed in order to assess changes in symptomatology and functional aspects before and after treatment and between T1 and T2. Z test was conducted, with a level of statistical significance *p* < 0.05. Confidence intervals were set at 95%.

### 2.5. Functional Characteristics of RA.DI.CA

#### 2.5.1. Design

**Figure 1:**
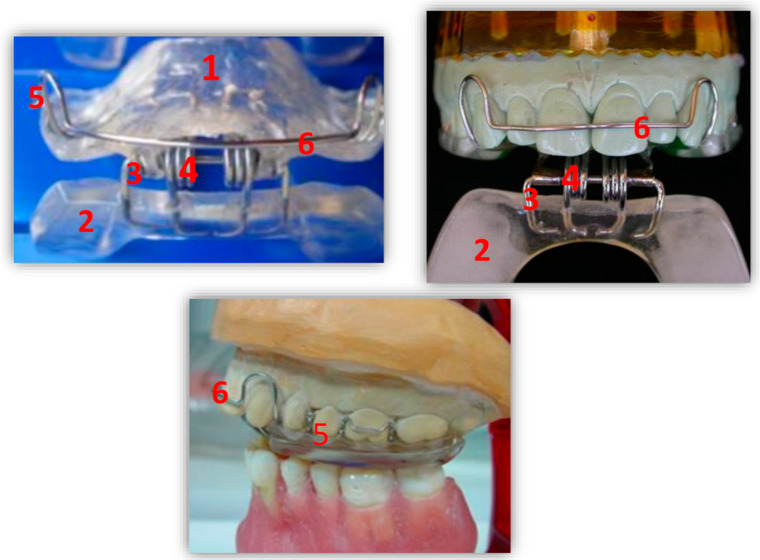
RA.DI.CA. splint and its components.

The device is composed as follows by (Figure 1):

A heat-cured acrylic resin upper plate (**1**),
A heat-cured acrylic resin lower plate (**2**),
An anterior hinge (**3**),
Two vestibular springs made with orthodontic wire (**4**),
Two or more Adams clasps and/or two ball clasps (**5**),
A vestibular steel arch (**6**).

The upper plate has two surfaces. The inner one also called “occlusal/palatal” adapts to the masticatory surfaces of the upper teeth and with the anterior 2/3 of the palatal vault. The outer one is smooth and faces the lower plate. Two Adams clasps are placed on teeth 1.6 and 2.6 and the ball ones are placed in the interdental space of 1.4–1.5 and 2.4–2.5. The upper plate plays a stabilizing role, having a double mucous and dental anchorage.

The lower plate, also shaped like a horseshoe, has two smooth surfaces. One is in contact with the outer surface of the upper plate and the other one with the masticatory surfaces of the lower teeth. The latter should be shaped in accordance with Spee and Wilson curves.

**Figure 2:**
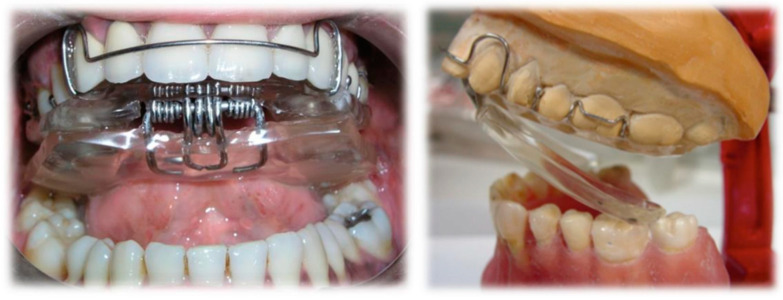
RA.DI.CA., mechanism of action (frontal and lateral view).

The two plates are connected to each other by a front hinge placed at the incisor level and by two (left and right) vestibular springs, connected in the canine-premolar site, in order to give an elastic resistance to the lower plate during the functional movements of mouth closure. The springs can be hard or soft according to the characteristics of the patient. The functional action is carried out through a “pushing” mechanism at the level of the posterior area of the lower dental arch, which induces a downward and forward movement of the mandibular condyle (Figure 2).

Therefore, by means of its components, the illustrated device has, as its main therapeutic goal, the recovery of the functional relationship between articular disc and mandibular condyle and in more severe cases, where the recovery is not achievable, the rehabilitation of jaw function and the reduction of algic symptomatology [4,5].

#### 2.5.2. Application Protocol of RA.DI.CA Splint

The management protocol included progressive phases which modified the duration of daily application of the occlusal splint, up to a minimum use.

It lasted not less than 6 weeks and no more than 24 months.

In general, at the end of therapy with the RA.DI.CA. splint, the patient is evaluated again, in order to establish the need to replace this kind of splint with other ones, such as DI.TRA (Direct Tridimensional Repositioning Appliance) or Michigan splint [18]. In this phase, the rationale was to guide the stabilization of the functional outcome.

## 3. Results

### 3.1. T0–Baseline

The sample was composed by 121 females (86.5 %) and 19 males (13.5%), with a mean age of 37 years (range 25–60).

Forty-one percent of patients (58 patients) had Class I dental occlusion, 38% (53 patients) had II Class dental occlusion, 9% (13 patients) had Class III dental occlusion, 12% (17 patients) reported a combination of the previous ones.

Fifty-six percent of the sample (78 patients) reported direct and indirect traumas, such as whiplash, falls, accidents at the medical history (except for the ones who caused mandibular and/or condylar fractures, which were exclusion criteria of the study, as mentioned in the previous section).

In the examined sample, 35% (49 patients) came to visit 6 months after the beginning of dysfunctional symptomatology, 35% (49 patients) 7 months to 1 year after, 30% (43 patients) over 3 years after.

In 60 % (84 patients), disc displacement affected right TMJ, in 40% (57 patients) it affected left TMJ. As for the articular symptomatology, the following characteristics were found: pain in the affected joint, n = 92 (65%); pain in the non-affected joint, n = 15 (11%); bilateral pain, n = 34 (24%). No patient had mild temporomandibular pain, 37% of the sample (52 patients) had moderate pain, 63% of the sample (88 patients) had severe pain.

As for comorbidities, headache in the temporal region, modified or provoked by oral function was present in 75% of sample (105 patients), of whom 53% (56 patients) had moderate headache and 47% (49 patients) had severe headache. Neck pain was detected in 65% of the sample (91patients), of whom 46% (42 patients) had moderate pain and 54% (49 patients) had severe pain.

As for the functional aspects, the following findings were reported: all the patients reported mouth opening limitation (< 35mm) and limitation of lateral excursion (<6 mm) on the affected side. The deviation of the mandibular movement towards the affected side during mouth opening was detected in 100%.

### 3.2. T1—After Treatment

#### 3.2.1. Effectiveness of RADICA Splint Therapy

**Figure 3:**
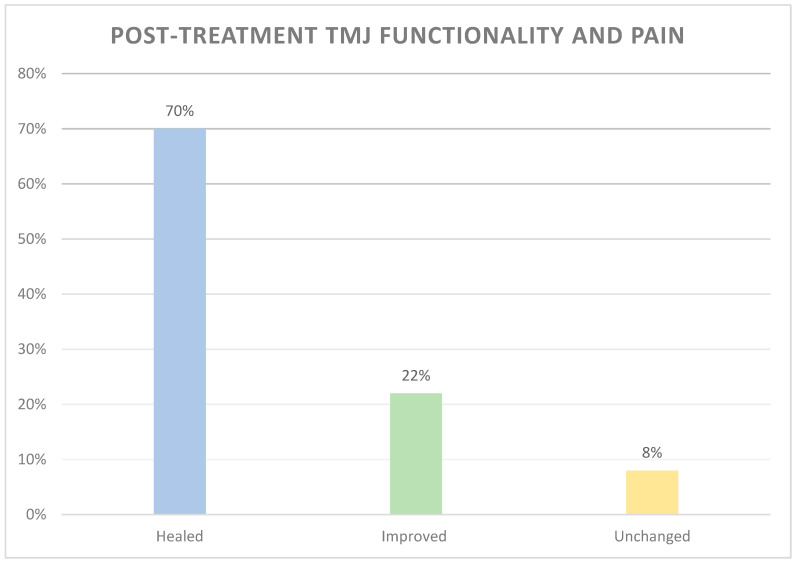
Results of treatment with the RA.DI.CA. splint at T1. (%) Patients for each category.

As for the results of treatment in terms of improvement of joint functionality and pain, the categories with the largest number of patients were “Healed” and “Improved”. Eleven patients (8%) did not report any improvement and no patient suffered a worsening of painful symptomatology and limitation (Figure 3).

As for mean values (at verbal numeric scale) of pain symptomatology (joint pain, neck pain, and headache) and range of mouth opening (in mm), the comparison between the ones before and after treatment was statistically significant (*p* < 0.05) in all the parameters analyzed (Table 1).

Furthermore, 92% of patients (130 subjects) reported no deviation in mandibular movement during the opening of the mouth and no limitation in lateral and protrusive excursions.

As for the perception of occlusal changes, only 7 patients (5%) reported a negative occlusal change after three months of active therapy. However, at T1, no one reported negative occlusal changes.

#### 3.2.2. Efficiency of RADICA Splint Therapy (Duration of Therapy)

The shortest therapy had a duration of 3 months and the longest one of 23 months with a mean duration of 15 ± 5 months. The functional recovery was obtained in a shorter time compared to the decrease of algic symptomatology (Table 2).

The number of patients who agreed to undergo a post-therapy MRI was 42 (38.8%).

The 42 patients who underwent the post-treatment MRI showed the following results:

- 10 patients (23.8%) gained a complete condyle-disc ratio.
- 19 patients (45.2%) gained a partial condyle-disc ratio.
- 13 patients (30.9%) did not gain the condyle-disc ratio.

### 3.3. T2–Follow-Up

The average follow-up time was 5.6 years (range 2–10 years).

There was not a statistically significant difference between T1 and T2, as for records analyzed (*p* > 0.05). The treatment outcome, achieved at the end of the active therapy (T1), remained stable (no significant improvements or worsening).

## 4. Discussion

In this study, the authors shared their experience using the RA.DI.CA. splint for the management of temporomandibular joint disc displacement without reduction, as the most common cause of jaw functional limitation. The device derives from a deepening of knowledge of the anatomical and physio-pathological aspects of the stomatognathic apparatus, supported by clinical experience and critical analysis of the therapeutic options proposed in the scientific literature. The need for finding a specific device for jaw functional limitation, aiming at standardizing the therapeutic protocols, regardless of the operator skills, guided the scientific research. In fact, in the past and even today, standard treatment protocols included the manipulation of the mandible to recapture the dislocated disc as elective therapy, mostly useful if applied earlier after the onset of symptoms [23,24,25], however such technique had a variability in the patient’s individual response and depends on the operators’ skills to a large extent. Furthermore, the idea was to find a therapy that was easy to implement by the patient, with a good degree of effectiveness and stability over time, along the lines of the treatments that the scientific literature already proposed. A therapeutic flowchart for TMDs, and among these for ADDwOR and jaw limitation, supported by evidence and based on diagnostic and clinical data, has been already proposed by the authors in previous studies [4,5,18,19,20]. Although this research does not have the purpose to be a comparative study, the authors considered it necessary to report an examination of the alternative treatment solutions for the management of ADDwOR.

As for the occlusal appliances, in the scientific community, there is no common thought about what type of device should be used in patients with jaw limitation and, in particular, with ADDwoR. There is a huge variety of splints with a targeted action on this type of pathology and many of these devices share a common action, each one with its own characteristics. As mentioned in the introduction, there are three types of occlusal splints which are commonly used in ADDwoR: the stabilization, the distraction/pivot, and the anterior repositioning splints. As for the stabilization splint, according to some authors [10,16,17], the main action is the increase of vertical dimension, which leads to a significant reduction of symptoms of closed lock. Other authors have reported less significant results [26,27]. As for the pivot splints are concerned, there are several types. Some authors suggest the use of the occlusal pivot appliances, as described by Sears [28], with an action similar to the distraction splints [29]. Compared to the previous type of splint, the distraction/pivot splints are supposed to have the additional benefit of mobilizing the condyle with a greater effect of condylar distraction and possibility of reducing joint disc [30]. However, which kind of splint has a greater effectiveness is a debated issue among researchers, as emerged from the study of Schmitter et al. [11] which reported that the stabilization splint seems to be more effective than the distraction splint in the closed lock therapy. In fact, these last authors maintained that the outcome of the splint therapy is achieved not by improving the disc position, but instead it is strongly related to the basic decoupling of neuromuscular reflex mechanism and reduction of TMJ stress [31,32]. On the contrary, according to Lundh et al., the therapeutic effect of flat occlusal splints (stabilization splints) was observed in 32% of patients. The same authors presented evidence that there is no significant difference between outcome of flat splint therapy and nontreatment group of patients [33].

The authors of this study, on their end, believe that a proper functionality and the absence of pain could remain stable over time only aiming, as much as possible, at the structural and positional recovery of the stomatognathic components (joint and muscular ones). In these circumstances, the goal of the treatment should be to recreate function by re-establishing the disc condyle relationship or, more often, by reducing restriction in movement, also in order to eliminate pain [31].

Two of the best-known distraction splints are the Functional Appliance by Rocabado, designed for joint distraction [34,35], and its modified version, MFDA (Modified Functional Distraction Appliance) by Festa [36]. In previous studies [19,20], compared to such devices, the RA.DI.CA. splint has proved to allow a greater amount of distraction, thanks to its design and type of springs used. Furthermore, it is easy-to-use with a minimum encumbrance for the patient, there were no occlusal changes perceived after treatment and, as for painful symptoms, there are more effective and long-lasting results [4,5].

As for the anterior repositioning splints, the aim is to restore the physiological disc-condyle relationship. However, the stability of disc recapturing depends on the range of disc displacement and the stage of internal derangement in TMJ. In cases of persistent closed lock, protrusive occlusal splint does not lead to the recapturing of the disc, but it has only a pain-relieving effect [37,38].

The RA.DI.CA. device acts as an “intra-oral physiotherapy”. By the induction of a downward and forward movement of the mandibular condyle, an “increase” of the intra-articular virtual spaces is allowed, resulting in a reduction of the pressure load on the retrodiscal (bilaminar) structures and the increase of blood flow and tissue tropism [4,5]. In fact, in this study, 99 out of 141 patients declared themselves “healed” from jaw functional limitation with no residual painful symptoms, therefore 130 patients (92%) obtained benefits from the application of the RA.DI.CA. splint, with a significant functional and symptomatic recovery, stable over time, in the follow-up. The stability of the results over time, compared to other treatments, is the most significant outcome. In fact, for example, as for physical therapy, the improvement is recorded only during the initial treatment session [39,40].

In patients with disc displacement without reduction and chronic dysfunction, the use of this device creates the conditions to recover spacial and, above all, functional relationship between condyle and disc. As confirmed by MRI also without recapturing the displaced disc, normal function could be obtained through the recovery of the functional relationship of the disc-condyle complex. The authors, aware that the recovery of the dislocated disc could not always be possible, also considering the initial conditions of the patient, have always focused on the recovery of joint mobility and functionality without pain. Despite this, post-treatment MRI also showed that in most patients who had undergone this evaluation, a complete or partial recovery of the dislocated disc was achieved [41,42,43].

In many patients with permanent disc displacement without reduction, restitutio ad integrum is not achievable, but nevertheless, the device has proved to be capable of performing functional and symptomatic recovery. Furthermore, this device allows an active and passive gymnastic with a gradual action, in order to recover not only the articular functionality but also the muscular one, made possible by the activation of the sliding of the myofibrils of actina and myosin in sarcomers, switched off by the contracture status [4,5]. The results of these combined actions on the symptomatic aspect were also noticed on the comorbidities associated to ADDwoR, such as neck pain and headache, with a statistical significance after treatment.

Overall, the recovery of functionality is achieved through the biomechanical repositioning of the stomatognathic components with the resulting restoration of normal mandibular range of motion, and through the reduction of pain, due to the biological and histological healing of the structural components.

As for surgical options, arthrocentesis and arthroplasty are among the suggested therapies in the scientific literature, with a high standard of effectiveness. Nevertheless, no significant differences between conservative and surgical treatment strategies were found [44] so, with the same results, non-surgical treatment should be recommended for TMJ closed lock before considering surgery.

Potential limitations of the study: bias after trial in data analysis were avoided by blinding the operators as for the type of splint used by the patients, during post-treatment and follow-up pain and functionality assessment.

## 5. Conclusions

The benefits of RA.DI.CA splint therapy are: (a) the effectiveness in terms of recovery of function and symptoms, also in the medium-long term follow up; (b) a low rate of adverse events; (c) a good tolerability and comfort for the patient. The recovery of the functional-orthopedic aspect is what allows also the recovery of symptoms and the stability of the patient’s conditions over time.

## Figures and Tables

**Table 1:** Changes in pain symptomatology (according to verbal numeric scale) and mouth opening (in mm) before and after treatment with Wilcoxon test and Z-test, *p* < 0.05. Bold type = statistically significant.

Parameter	Before Treatment (Mean ± SD)	Before Treatment (Median)	After Treatment T1 (Mean ± SD)	After Treatment T1 (Median)	Wilcoxon Test (W)	Z Test Value	*p* Value
**Joint pain**	63 ± 10.67	63	8.32 ± 13.12	6	8385	8.72	**<0.0001**
**Neck pain**	59.67 ± 13.52	61	20.09 ± 21.10	15	3741	7.40	**<0.0001**
**Headache**	60.40 ± 16.54	62	13.3 ± 20.63	0	5565	8.89	**<0.0001**
**Mouth opening**	30.27 ± 4.45	30	41.44 ± 5.44	40	9870	10.26	**<0.0001**

**Table 2:** Average time of recovery (in months).

Study Variable	Average Time of Recovery (in Months)
**Jaw Limitation**	3.8 ± 1.2
**Tmd Pain**	6.4 ± 1.7
**Neck Pain**	8.1 ± 1.8
**Headache**	9.6 ± 2.4

## Data Availability

The authors confirm that the data supporting the findings of the study are available within the article.

## References

[1] Cooper B.C., Kleinberg I. (2007). Examination of a large patient population for the presence of symptoms and signs of temporomandibular disorders. CRANIO^®^.

[2] Miernik M., Więckiewicz W. (2015). The Basic Conservative Treatment of Temporomandibular Joint Anterior Disc Displacement Without Reduction—Review. Adv. Clin. Exp. Med..

[3] Dworkin S.F., LeResche L. (1992). Research diagnostic criteria for temporomandibular disorders: Review, criteria, examinations and specifications, critique. J. Craniomandib. Disord..

[4] Di Paolo C., Rampello A. (1993). Lock intrarticolare dell’ATM. Terapia non chirurgica. Dent. Cadmos.

[5] Cascone P., Di Paolo C., Dall’eziopatogenesi alla terapia (2004). Patologia dell’Articolazione Temporomandibolare.

[6] Schiffman E., Ohrbach R., Truelove E., Look J., Anderson G., Goulet J.-P., List T., Svensson P., Gonzalez Y., Lobbezoo F. (2014). Diagnostic Criteria for Temporomandibular Disorders (DC/TMD) for Clinical and Research Applications: Recommendations of the International RDC/TMD Consortium Network and Orofacial Pain Special Interest Group. J. Oral Facial Pain Headache.

[7] McNeill C. (1990). Craniomandibular Disorders Guidelines for Evaluation Diagnosis and Management: The American Academy of Craniomandibular Disorders.

[8] Yura S. (2012). Natural course of acute closed lock of the temporomandibular joint. Br. J. Oral Maxillofac. Surg..

[9] Dimitroulis G. (2005). The prevalence of osteoarthrosis in cases of advanced internal derangement of the Temporomandibular Joint: A clinical, surgical and histological study. Int. J. Oral Maxillofac. Surg..

[10] Stiesch-Scholz M., Tschernitschek H., Rossbach A. (2002). Early begin of splint therapy improves treatment outcome in patients with temporomandibular joint disc displacement without reduction. Clin. Oral Investig..

[11] Schmitter M., Zahran M., Duc J.-M., Henschel V., Rammelsberg P. (2005). Conservative Therapy in Patients with Anterior Disc Displacement without Reduction Using 2 Common Splints: A Randomized Clinical Trial. J. Oral Maxillofac. Surg..

[12] Zotti F., Albanese M., Rodella L.F., Nocini P.F. (2019). Platelet-Rich Plasma in Treatment of Temporomandibular Joint Dysfunctions: Narrative Review. Int. J. Mol. Sci..

[13] Al-Delayme R.M.A., Alnuamy S.H., Hamid F.T., Azzamily T.J., Ismaeel S.A., Sammir R., Hadeel M., Nabeel J., Shwan R., Alfalahi S.J. (2017). The Efficacy of Platelets Rich Plasma Injection in the Superior Joint Space of the Temporomandibular Joint Guided by Ultra Sound in Patients with Non-reducing Disk Displacement. J. Maxillofac. Oral Surg..

[14] Kurina H., Kurashina K., Ohtsuka A. (1999). Efficacy of mandibular manipulation technique in reducing the permanently displaced temporomandibular joint disc. J. Oral Maxillofac. Surg..

[15] Dimitroulis G. (2018). Management of temporomandibular joint disorders: A surgeon’s perspective. Aust. Dent. J..

[16] Haketa T., Kino K., Sugisaki M., Takaoka M., Ohta T. (2010). Randomized Clinical Trial of Treatment for TMJ Disc Displacement. J. Dent. Res..

[17] Shoji Y.N. (1995). Nonsurgical treatment of anterior disk displacement of the temporomandibular joint: A case report on the relationship between condylar rotation and translation. CRANIO^®^.

[18] Meshkova D.T., Di Giacomo P., Panti F., D’Urso A., Serritella E., Di Paolo C. (2019). Application of a Systematic Protocol in the Treatment of TMDs with Occlusal Appliances: Effectiveness and Efficiency in a Longitudinal Retrospective Study with Medium-Term Follow-Up. J. Int. Soc. Prev. Community Dent..

[19] Di Paolo C., Accivile E., Chimenti C., Carbonelli A. (1987). Electrognathography: Diagnostic adjunct in the treatment of closed-lock joint. Dent. Cadmos.

[20] Di Paolo C., Liberatore G.M., Rampello A., Panti F. (1994). A longitudinal analysis of dysfunctional pathology of the TMJ: An assessment of a sample of patients undergoing nonsurgical therapy. Minerva Stomatol..

[21] De Boever J.A., Carlsson G.E., Klineberg I.J. (2000). Review—Need for occlusal therapy and prosthodontic treatment in the management of temporomandibular disorders, Part II, Tooth loss and prosthodontic treatment. J. Oral Rehabil..

[22] Di Paolo C., Cascone P. (1998). Ruolo dei fattori occlusali nell’eziopatogenesi dei DTM—Considerazioni clinico-statistiche. Dent. Cadmos.

[23] Gotou M., Nagata K., Sugawara Y. (2010). Short-term effectiveness of the Jog- manipulation technique for temporomandibular disorders (TMD)patients with limited mouth opening—A randomized controlled trial. J. Jpn. Soc. TMJ.

[24] Tuncer A.B., Ergun N., Tuncer A.H., Karahan S. (2013). Effectiveness of manual therapy and home physical therapy in patients with temporomandibular disorders: A randomized controlled trial. J. Bodyw. Mov. Ther..

[25] Yoshida H., Fukumura Y., Suzuki S., Fujita S., Kenzo O., Yoshikado R., Nakagawa M., Inoue A., Sako J., Yamada K. (2005). Simple Manipulation Therapy for Temporomandibular joint Internal Derangement with Closed Lock. Asian J. Oral Maxillofac. Surg..

[26] Lee H.S., Baek H.S., Song D.S., Kim H.C., Kim H.G., Kim B.J., Kim M.S., Shin S.H., Jung S.H., Kim C.H. (2013). Effect of simultaneous therapy of arthrocentesis and occlusal splints on temporomandibular disorders: Anterior disc displacement without reduction. J. Korean Assoc. Oral Maxillofac. Surg..

[27] Kai S., Kai H., Tabata O., Shiratsuchi Y., Ohishi M. (1998). Long-term outcomes of nonsurgical treatment in nonreducing anteriorly displaced disk of the temporomandibular joint. Oral Surg. Oral Med. Oral Pathol. Oral Radiol. Endodontol..

[28] Sears V.H. (1956). Occlusal pivots. J. Prosthet. Dent..

[29] Moncayo S. (1994). Biomechanics of pivoting appliances. J. Orofac. Pain.

[30] Muhtarogullari M., Avci M., Yuzugullu B. (2014). Efficiency of pivot splints as jaw exercise apparatus in combination with stabilization splints in anterior disc displacement without reduction: A retrospective study. Head Face Med..

[31] Stiesch-Scholz M., Kempert J., Wolter S., Tschernitschek H., Rossbach A. (2005). Comparative study on splint therapy of anterior disc displacement without reduction. J. Oral Rehabil..

[32] Ohnuki T., Fukuda M., Nakata A., Nagai H., Takahashi T., Sasano T., Miyamoto Y. (2006). Evaluation of the position, mobility, and morphology of the disc by MRI before and after four different treatments for temporomandibular joint disorders. Dentomaxillofac. Radiol..

[33] Lundh H., Westesson P.L., Eriksson L., Brooks S.L. (1992). Temporomandibular joint disk displacement without reduction. Oral Surg. Oral Med. Oral Pathol..

[34] Rocabado M. (1984). Joint distraction with a functional maxillomandibular orthopedic appliance. J. Craniomandib. Pract..

[35] Festa F. (1985). Joint distraction and condyle advancement with a modified functional distraction appliance. CRANIO^®^.

[36] Festa F. (1995). Modified functional distraction appliance an appliance for chronic lock and ankylosis. CRANIO^®^.

[37] Eberhard D., Bantleon H.P., Steger W. (2002). The efficacy of anterior repositioning splint therapy studied by magnetic resonance imaging. Eur. J. Orthod..

[38] Simmons H.C., Gibbs S.J. (1995). Recapture of temporomandibular joint disks using anterior repositioning appliances: An MRI study. CRANIO^®^.

[39] Craane B., Dijkstra P.U., Stappaerts K., De Laat A. (2012). Randomized controlled trial on physical therapy for TMJ closed lock. J. Dent. Res..

[40] Nagata K., Hori S., Mizuhashi R., Yokoe T., Atsumi Y., Wataru N., Goto M. (2019). Efficacy of mandibular manipulation technique for temporomandibular disorders patients with mouth opening limitation: A randomized controlled trial for comparison with improved multimodal therapy. J. Prosthodont. Res..

[41] Choi B.H., Yoo J.H., Lee W.Y., Do K. (1994). Comparison of magnetic resonance imaging before and after nonsurgical treatment of closed lock. Oral Surg. Oral Med. Oral Pathol..

[42] Kirk W. (1991). Magnetic resonance imaging and tomographic evaluation of occlusal appliance treatment for advanced internal derangement of the temporomandibular joint. J. Oral Maxillofac. Surg..

[43] McNeill C. (1985). The optimum temporomandibular joint condyle position in clinical practice. Int. J. Periodont. Restor. Dent..

[44] Al-Baghdadi M., Durham J., Araujo-Soares V., Robalino S., Errington L., Steele J. (2014). TMJ disc displacement without reduction management: A systematic review. J. Dent. Res..

